# Microarray Analysis of mRNA and MicroRNA Expression Profile Reveals the Role of **β**-Sitosterol-D-glucoside in the Proliferation of Neural Stem Cell

**DOI:** 10.1155/2013/360302

**Published:** 2013-12-11

**Authors:** Li-hua Jiang, Nian-yun Yang, Xiao-lin Yuan, Yi-jie Zou, Ze-qun Jiang, Feng-ming Zhao, Jian-ping Chen, Ming-yan Wang, Da-xiang Lu

**Affiliations:** ^1^Medical College of Jinan University, 601 Huangpu Road West, Guangzhou 510632, China; ^2^Department of Pharmacognosy, Nanjing University of Chinese Medicine, Nanjing 210038, China; ^3^Basic Medical College of Nanjing University of Chinese Medicine, Nanjing 210038, China; ^4^Jiangsu Province Hospital of Traditional Chinese Medicine, Nanjing 210029, China

## Abstract

Neural stem cells (NSCs) are self-regenerating cells, but their regenerative capacity is limited. The present study was conducted to investigate the effect of **β**-sitosterol-D-glucoside (BSSG) on the proliferation of hippocampal NSCs and to determine the corresponding molecular mechanism. Results of CCK-8 assay showed that BSSG significantly increased NSC proliferation and the effectiveness of BSSG was similar to that of basic fibroblast growth factor and epidermal growth factor. mRNA expression profiling showed that 960 genes were differentially expressed after NSCs were treated with BSSG. Among the 960 genes, IGF1 is considered as a key regulatory gene that functionally promotes NSC proliferation. MicroRNA (miRNA) expression profiling indicated that 30 and 84 miRNAs were upregulated and downregulated, respectively. miRNA-mRNA relevance analysis revealed that numerous mRNAs including IGF1 mRNA were negatively regulated by miRNAs with decreased expression, thereby increasing the corresponding mRNA expression. The increased expression of IGF1 protein was validated by ELISA. Picropodophyllin (PPP, an inhibitor of IGF-1R) inhibition test confirmed that the proliferation-enhancing effect depended on IGF1. This study provided information about BSSG as an efficient and inexpensive growth factor alternative, of which the effect is closely involved in IGF1.

## 1. Introduction

Neural stem cells (NSCs) are defined as undifferentiated cells that have the ability of self-renewal and the potential to generate neurons, astrocytes, or oligodendrocytes in the central nervous system [1, 2]. NSCs are located in the subventricular zone (SVZ) and the subgranular zone (SGZ) of hippocampus. NSCs and neural regeneration have been extensively studied to provide proper treatment for encephalic diseases [3, 4] by cell transplantation. However, a majority of NSCs are arrested at the G0 phase of the mitotic cell cycle; such a limited regenerative capacity of endogenous and grafted NSCs is attributed to the inhibition of NSC proliferation and neurogenesis [5]. Studies have also suggested that several active components of herbs such as Tenuigenin [6] and mitogens such as epidermal growth factor (EGF) [7] and basic fibroblast growth factor (bFGF) [8] accelerate NSC proliferation. However, these active components are costly or chemically unstable, limiting their application. Therefore, reliable alternative chemical components should be identified.

In this study, the first confirmation that *β*-sitosterol-D-glucoside (BSSG, a sterolin) increases the proliferation of hippocampal NSCs was presented. Studies have showed multiple biological activity of BSSG, including anti-inflammatory effects [9, 10], anthelmintic activity [11], and immunomodulating activity [12, 13]. In the study, BSSG promoted NSC proliferation as a result of regulation of numerous genes, especially increasing IGF1 expression.

Recent advances in bioinformatics and high-throughput technologies such as microarray analysis are bringing about a revolution in our understanding of the molecular mechanisms underlying biological processes. In this study, mRNA and microRNA (miRNA) expression microarray analyses were performed to understand the molecular mechanism of the effect on NSCs. miRNAs are small endogenous, noncoding RNAs that are highly conserved and that have been recognized as a powerful tool for regulating gene expression through the RNA interference pathway [14, 15]. With the ability of one miRNA to bind and regulate numerous mRNAs and the potential for a single mRNA to be targeted by multiple miRNAs, it is possible to fine-tune the expression of proteins within the cell in a very precise manner [16].

Some analytical methods are applied in the microarray analysis, including Gene Ontology (GO) analysis. GO analysis (http://www.geneontology.org/) provides a controlled vocabulary to describe the gene and the gene product attributes in any organism. GO covers three domains: biological process, cellular component, and molecular function [17]. Biological process refers to a biological objective to which the gene or gene product contributes. A process is accomplished via one or more ordered assemblies of molecular functions. Molecular function is defined as the biochemical activity of a gene product. Cellular component refers to the place in the cell where a gene product is active. These terms reflect our understanding of eukaryotic cell structure. Among these domains, biological process helps understand the biological functions specifying where or when the event actually occurs. Thus, biological process was used to describe the specific biological functions of differentially expressed genes. In addition, genic network analysis helps understand the interacting genes.

## 2. Materials and Methods

### 2.1. Preparation of BSSG

BSSG (purity: 98%, provided by Department of Pharmacognosy, Nanjing University of Chinese Medicine) stock solution was prepared in Dimethyl Sulfoxide (DMSO). Before each experiment was performed, the solution was diluted in a fresh medium to obtain a final DMSO concentration of ≤0.1%.

### 2.2. Primary NSC Culture and Identification

Primary NSC culture was established according to a previously published protocol [18]. In brief, the hippocampus of a Sprague-Dawley rat embryo at 16 d of embryonic stage was dissected in cold CMF-HBSS and then dissociated mechanically. The cells were collected by centrifugation and resuspended in a neurobasal medium (Gibco, CA, USA) supplemented with epidermal growth factor (EGF, 20 ng/mL; PeproTech Inc., Rocky Hill, NJ, USA), basic fibroblast growth factor (bFGF, 20 ng/mL; PeproTech Inc. USA), B27 supplement (2%; Gibco, USA), penicillin (50 U/mL), and streptomycin (50 *μ*g/mL). The cells were adjusted to a density of 1 × 10^5^ cells/mL and planted in culture flasks. The medium was replaced with 1/2 of the same medium at an interval of 3 d. The cells were stained by immunocytochemistry with primary antibody against nestin (Boshide, Wuhan, China) to make the identification of NSCs.

### 2.3. CCK-8 Assay

#### 2.3.1. Dose-Dependent Cell Proliferation Detection

Dose-dependent cell viability was monitored using a cell counting kit-8 (CCK-8) assay. CCK-8 is a sensitive nonradioactive colorimetric assay used to determine the number of viable cells in cell proliferation and cytotoxicity assays. In the study, NSCs were cultured in 96-well plates containing the growth culture medium at a cell density of 5 × 10^3^ cells per well. The cells were divided into nine groups: control group and BSSG treatment groups (1.25, 2.5, 5, 10, 20, 40, 80, and 100 *μ*M BSSG). Each group was designed to establish six double-pore treatments. Each BSSG treatment group was treated with the corresponding amount of BSSG. The cells were treated for 72 h as described, whereafter CCK-8 solution (Beyotime Biotech, Haimen, China) was added to the cell culture medium to a final concentration of 10 *μ*L/100 *μ*L and incubated for another 4 h at 37°C. Absorbance was measured at 450 nm to determine cell viability as a percentage, using a microplate reader (ELx800, BioTek Instruments, Inc., Winooski, VT, USA).

#### 2.3.2. Comparison of NSC Proliferation Promoted by BSSG, bFGF, and EGF

To understand the effectiveness of BSSG on enhancing NSCs proliferation, we compared BSSG with bFGF and EGF by performing CCK-8 assay. The cells were cultured in 96-well plates at a cell density of 1 × 10^4^ cells per well and then divided into six treatment groups: bFGF and EGF vacancy group (bFGF^−^EGF^−^: with neurobasal medium and B27 supplement); bFGF group (with neurobasal medium, B27 supplement, and bFGF 20 ng/mL); EGF group (with neurobasal medium, B27 supplement, and EGF 20 ng/mL); and bFGF^−^EGF^−^ + BSSG groups (10, 20, and 40 *μ*M). The cells were treated for 72 h as described above, and CCK-8 assay was performed.

### 2.4. mRNA Expression Microarray Analysis

#### 2.4.1. mRNA Expression Profiling

mRNA expression microarray analysis was performed by use of Roche-NimbleGen Rattus norvegicus 12 × 135 K Array (Roche, supplied by KangChen Corp), in order to understand the effect of BSSG on regulation of mRNA, disclosing the mechanism of BSSG promoting NSCs proliferation.

Total RNA was obtained from each sample (five samples from the control group and five samples from the BSSG-treated group (40 *μ*M), the cells were treated for 72 h as described above) and quantified by NanoDrop ND-1000. The total RNA was used for labeling and array hybridization based on the following steps: (1) reverse transcription using superscript ds-cDNA synthesis kit (Invitrogen, USA); (2) ds-cDNA labeling using one-color DNA labeling kit (Roche NimbleGen, USA); (3) array hybridization using NimbleGen hybridization system and washed using NimbleGen wash buffer kit; and (4) array scanning using the Axon GenePix 4000B microarray scanner (Molecular Devices Corporation, Sunnyvale, CA, USA).

The scanned images were imported into the NimbleScan software (version 2.6) for grid alignment and expression data analysis. The expression data were normalized by quartile normalization and robust multichip average (RMA) algorithm included in the NimbleScan software. The probe level files and the gene level files were generated after normalization. The ten gene level files were imported into Agilent GeneSpring GX software (version 11.5.1) for further analysis. Differentially expressed genes were identified by volcano plot filtering (*P* value < 0.05; fold change ≥2.0 or ≤0.5).

The genic network was plotted by use of the search tool STRING (http://string-db.org/) and drawing tool cytoscape. Then the connectivity analysis of the network was performed to obtain the “hubs” of the gene nodes, using one-side Fisher's exact test [19]. Network “nodes” (or “components”) represent genes. Nodes with large degree values are commonly referred to as “hubs.” The hub genes were considered as the important genes, which play significant roles in the functions and structure of cells.

#### 2.4.2. Real-Time PCR Validation for mRNA Expression Profiling

Real-time PCR was performed to validate the mRNA expression profiling obtained. Total RNA (obtained from the same samples as mentioned in mRNA microarray analysis) was reverse transcribed with SuperScript II reverse transcriptase (Invitrogen). Real-time PCR was performed using the ABI PRISM7900 system (Applied Biosystems, Foster City, CA, USA), in the presence of forward and reverse primers for the target genes, or forward and reverse primers for the GAPDH gene used as reference. Relative quantification of the target gene is determined by calculating the ratio between the concentration of the target gene and that of the reference.

The primer sequences of the GAPDH gene and the target genes are listed in Table 1.

### 2.5. miRNA Microarray Analysis

#### 2.5.1. miRNA Expression Profiling

Evidence showed the important functions of miRNAs in stem cell regulation [20, 21]. Specific miRNAs modulate the functions of many types of stem cells, including neural stem/progenitor cells [22, 23]. In the present study, the miRNA expression profiling was analyzed to understand the effect of BSSG on regulation of miRNA, describing the correlation of miRNA and mRNA. In brief, total RNA (obtained from the same samples as mentioned in mRNA microarray analysis) was harvested using TRIZOL (Invitrogen) and miRNeasy mini kit (QIAGEN) according to the manufacturer's instructions. After the total RNA was measured using NanoDrop 1000, the samples were labeled using the miRCURY Hy3/Hy5 power labeling kit and hybridized in miRCURY LNA Array (v.18.0). Following the washing steps, the slides were scanned by the Agilent Scanner G2505C.

The scanned images were imported into GenePix Pro 6.0 software (Axon) for grid alignment and data extraction. The replicated miRNAs were averaged and the miRNAs with intensities ≥30 in all of the samples were chosen to calculate the normalization factor. The expressed data were normalized by median normalization. After normalization, significantly and differentially expressed miRNAs were identified by volcano plot filtering.

#### 2.5.2. Real-Time PCR Validation for miRNA Expression Profiling

Real-time PCR was performed to validate the differential miRNA expression profiling obtained. Total RNA was reverse-transcribed to cDNA using AMV reverse transcriptase (Epicentre), RNase (Epicentre), dNTP (HyTest Ltd), RT buffer, and RT primers (Invitrogen). The mixture was incubated at 16°C for 30 min, 42°C for 40 min, and 85°C for 5 min to generate a library of miRNA cDNAs. U6 is used as an internal control for normalization. Real-time PCR was subsequently performed using an ABI PRISM7900 system (Applied Biosystems, Foster City, CA, USA) according to a standardized protocol. The reactions were incubated at 95°C for 10 min, followed by 40 cycles at an interval of 10 s at 95°C and an interval of 1 min at 60°C. Data were analyzed by 2^−ΔΔCT^. The primer sequences of the internal control gene and the target genes are listed in Tables 2 and 3.

### 2.6. IGF1 Protein Determination

IGF1 protein determination were performed to investigate the key regulator by which BSSG promotes NSC proliferation. The cells were cultured in 24-well plates at a cell density of 5 × 10^4^ cells per well and divided into four groups: a control group and BSSG-treated groups (10, 20, and 40 *μ*M). Each group was designed to establish six double-pore treatments. The cells were treated for 72 h as described above. The supernatant was collected and processed using the Quantikine rat IGF1 ELISA kit (R&D Systems, Inc., Minneapolis, MN, USA), following the instructions provided by the manufacturer. The absorbance of each well was determined using a microplate reader (EL×800, BioTek instruments, Inc., Winooski, VT, USA) set at 450 nm; the readings at 570 nm were subtracted from the readings at 450 nm. The quantity of cells in each well was counted using a cell counting plate. IGF1 levels were reported in pg/10,000 cells.

### 2.7. PPP Inhibition Test

IGF1 is mediated by type 1 IGF receptor (IGF1R). PPP is an inhibitor of IGF1R [24]. The inhibitory effect of PPP on IGF1R did not coinhibit the insulin receptor (IR) or compete with ATP in vitro kinase assays, suggesting that PPP may inhibit IGF1R autophosphorylation at the substrate level [25]. If PPP prevents BSSG-induced NSC proliferation, then the function of BSSG depends on IGF1.

In the study, PPP (Tocris Bioscience, Bristol, UK) stock solutions were prepared in DMSO and stored at 4°C. Before each experiment was performed, these solutions were diluted in a fresh medium to obtain a final DMSO concentration of <0.1%. The cells were cultured in 96-well plates at a cell density of 1 × 10^4^ cells per well and divided among the PPP-BSSG treated groups (pair-wise; with PPP doses of 0, 0.01, 0.1, 1, and 2 *μ*M and BSSG doses of 0, 10, 20, and 40 *μ*M). Each group was designed to establish four double-pore experiments. The cells were treated for 72 h, and CCK-8 assay was performed. Absorbance was measured at 450 nm to determine cell viability as a percentage.

### 2.8. Statistical Analyses

Statistical analyses were performed by use of SPSS version 16.0 software program for windows (SPSS, Inc., Chicago, IL). Multiple comparisons were made using one-way ANOVA, followed by the Bonferroni posttest. All data are presented as Mean ± SD, and statistical significance was accepted at the 5% level.

## 3. Results

### 3.1. Identification of NSCs

**Figure 1:**
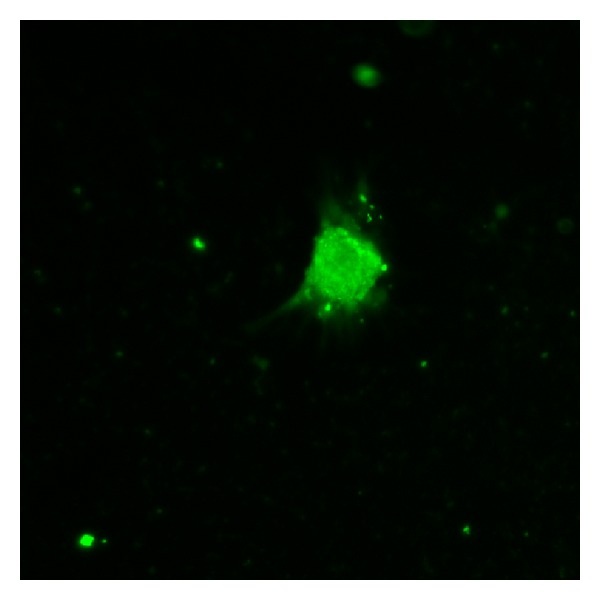
Identification of NSCs. Cultured NSCs were stained by immunocytochemistry with primary antibody against nestin (original magnification: 100x).

NSCs were positive for nestin (Figure 1).

### 3.2. Dose-Dependent Cell Proliferation Detection

**Figure 2:**
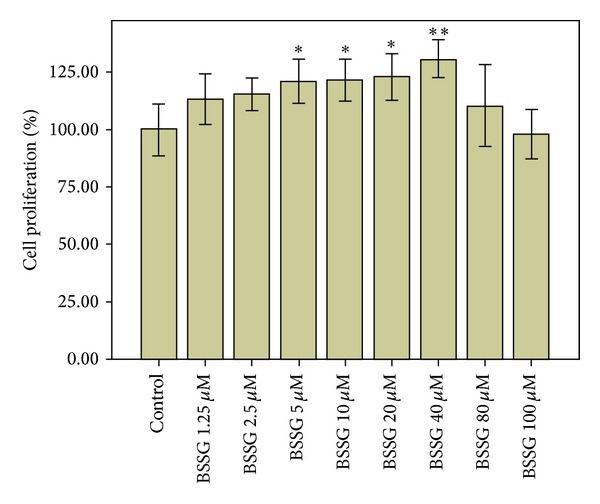
Dose-dependent effects of BSSG on cell proliferation. Dose-dependent effects of BSSG on cell proliferation were determined by CCK-8 assay and the data are plotted as percentages of control cell proliferation. Data are presented as Mean ± SD (*n* = 6); *P < 0.05 and **P < 0.01 compared with the control. BSSG significantly increased cell proliferation percentage at a concentration of 40 *μ*M (*P* < 0.01) and at concentrations of 5, 10, and 20 *μ*M (*P* < 0.05).

BSSG significantly increased NSC proliferation at concentration of 40 *μ*M (*P* < 0.01). The same result was observed at 5, 10, and 20 *μ*M BSSG (*P* < 0.05, Figure 2).

### 3.3. Comparison of NSC Proliferation Promoted by BSSG, BFGF, and EGF

**Figure 3:**
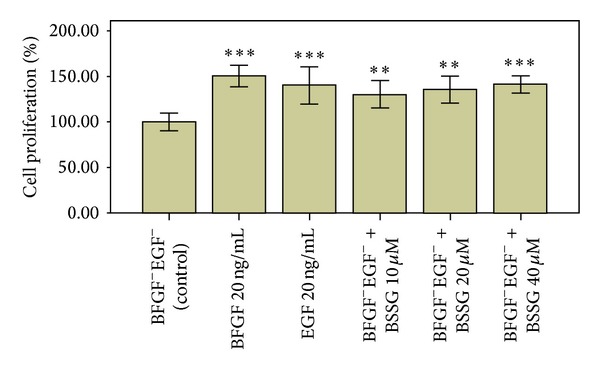
Comparison of NSC Proliferation Promoted by BSSG, BFGF, and EGF. Comparison of NSC proliferation promoted by BSSG, bFGF and EGF was determined by CCK-8 assay and the data are plotted as percentages of control cell proliferation. Data are presented as Mean ± SD (*n* = 6); **P < 0.01, ***P < 0.001 compared with the control. NSCs proliferation was induced by adding bFGF (20 ng/mL, *P* < 0.001), EGF (20 ng/mL; *P* < 0.001), and BSSG at different concentrations (10 and 20 *μ*M, *P* < 0.01; 40 *μ*M, *P* < 0.001). The effect of BSSG on NSC proliferation at 40 *μ*M was similar to those of bFGF and EGF.

NSCs proliferation was induced by adding bFGF (20 ng/mL; *P* < 0.001), EGF (20 ng/mL; *P* < 0.001), and BSSG at different concentrations (10 and 20 *μ*M, *P* < 0.01; 40 *μ*M, *P* < 0.001). The effect of BSSG on NSC proliferation at 40 *μ*M was similar to those of bFGF and EGF (Figure 3).

### 3.4. mRNA Expression Profiling

The result of mRNA expression microarray analysis showed that 960 genes were differentially expressed after NSCs were treated with BSSG, including 333 upregulation genes (fold change ≥2) and 627 downregulation genes (fold change ≤0.5). The differential expression genes were described using GO term analysis (biological process). The main differentially expressed genes are listed in Tables 4 and 5.

Tables 4 and 5 revealed that the majority of upregulation genes were involved in mitotic cell cycle particularly in the M phase, enhancing cell proliferation. By contrast, the majority of the downregulation genes were involved in cell differentiation, indicating that cell differentiation was inhibited, and accordingly, more possibility of cell proliferation was afforded.

### 3.5. Genic Network Analysis

**Figure 4:**
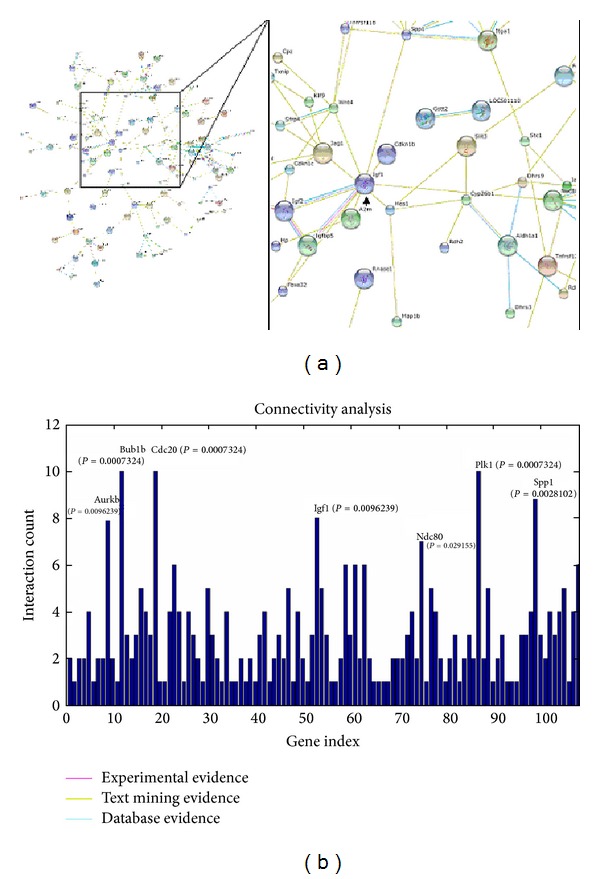
Genic network analysis. (a) Genic network. The genic network was plotted by use of the search tool STRING and drawing tool cytoscape to understand the interacting genes. The genic network consists of the majority of the upregulated and downregulated genes. The integral network and its magnified image are shown on the left and right parts, respectively. IGF1 is indicated by an arrow. (b) Connectivity analysis on the network. The “hubs” of the gene nodes were determined by interaction count and *P* value; thus, Bub1b, Cdc20, 1Plk1, Spp1, IGF1, Aurkb, and Ndc80 are the hub genes.

The genic network consists of the majority of the upregulation and downregulation genes (Figure 4(a)). The “hubs” of the gene nodes in the genic network include Bub1b (budding uninhibited by benzimidazoles 1 homolog, beta), Cdc20 (cell division cycle 20 homolog), Plk1 (polo-like kinase 1), Spp1 (Sialoprotein/osteopontin), IGF1 (insulin-like growth factor I), Aurkb (aurora kinase B), and Ndc80 (kinetochore associated 2) based on the results of connectivity analysis (Figure 4(b)). BSSG increased the expression of the hub genes, which are mostly involved in cell cycle (the functions of these genes see Genecards, http://www.genecards.org/), thereby enhancing NSC proliferation.

### 3.6. Real-Time PCR Validation for mRNA Expression Profiling

The mRNA expression profiling was validated by real-time PCR. The comparison of quantified mRNA expressions obtained using real-time PCR and microarray analysis was performed to determine the reliability of microarray analysis (Table 6). Table 6 showed that the fold changes (test versus control) of microarray analysis were almost similar to those of PCR; an increase or a decrease in gene expression quantified by microarray analysis was consistent with the result of PCR. This result indicated that the result of the microarray analysis was reliable.

### 3.7. miRNA Expression Profiling

**Figure 5:**
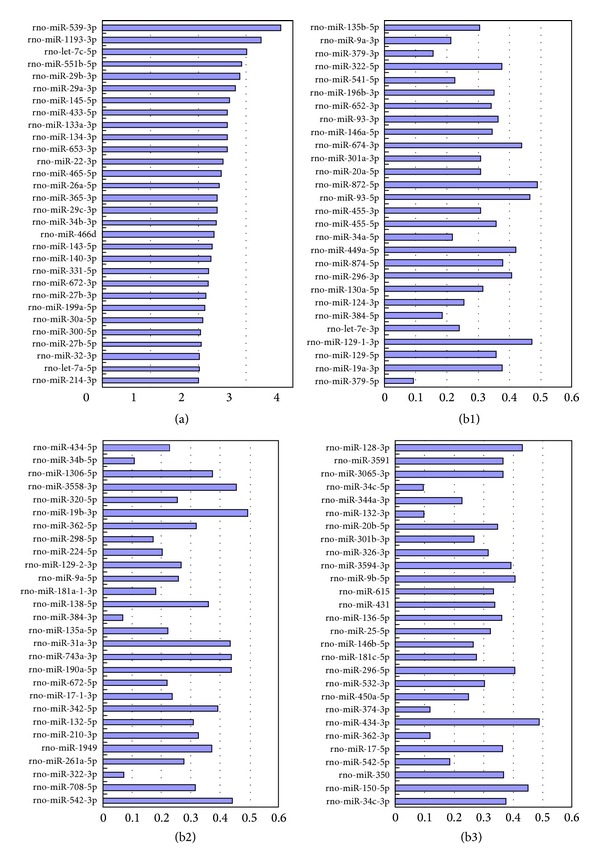
Differentially expressed miRNA. *Y*-axis shows the differentially expressed mRNAs; *X*-axis represents the fold change (test versus control), or the ratio of miRNA expression in the BSSG-treated group (test group) to the miRNA expression in the control group. (a) Upregulation miRNA; (b1), (b2), and (b3): downregulation miRNA.

A total of 30 upregulated miRNAs (test versus control, fold change ≥2) and 84 downregulated miRNAs (test versus control, fold change ≤0.5) were obtained after the NSCs were treated with BSSG (Figure 5).

### 3.8. Real-Time PCR Validation for miRNA Microarray Analysis

The miRNA expression profiling was validated by real-time PCR. The comparison of quantified miRNA expressions obtained using real-time PCR and microarray analysis was performed to determine the reliability of microarray analysis (Table 7). Table 7 showed that the fold changes (test versus control) obtained by microarray analysis were almost similar to those obtained by PCR. An increase or a decrease in gene expression quantified by microarray analysis was consistent with the result of PCR. This result indicated that the result of microarray analysis was reliable.

### 3.9. miRNA-mRNA Correlation Analysis

**Figure 6:**
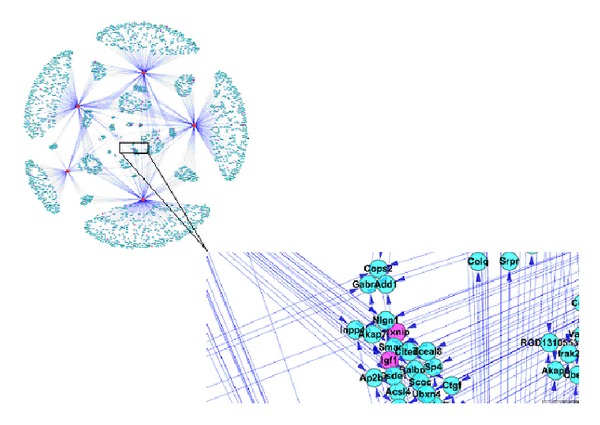
miRNA-mRNA Interactome Network. The integral chart and its magnified image were shown on the upper left and lower right corners, respectively. Red square nodes represented the five miRNAs: miR-322-5p; miR-301a-3p; miR-129-5p; miR-322-3p, and miR-129-2-3p. Turquoise round nodes represented the target genes of these miRNAs. Pink round nodes represented the upregulated mRNAs correlated with these miRNAs; green round nodes represented the downregulated mRNAs. The network revealed that numerous mRNAs were regulated by the miRNAs and the expressions of many mRNAs including IGF1 were increased.

miRNAs are recognized as a powerful tool used to regulate gene expression via the RNA interference pathway [14, 15]. Thus, miRNA-mRNA correlation analysis was carried out to further understand the effect of BSSG on regulating NSCs genes. Five downregulated miRNAs, miR-322-5p, miR-301a-3p, miR-129-5p, miR-322-3p, and miR-129-2-3p, of which the target mRNAs are involved in regulation of cell proliferation (based on miRWalk database http://www.ma.uni-heidelberg.de/apps/zmf/mirwalk/ and DAVID Bioinformatics Resources 6.7 http://david.abcc.ncifcrf.gov/tools.jsp), were selected to perform correlation analysis. The interactions between the five miRNAs and predicted target mRNAs (included in the differential expression genes obtained by the mRNA microarray analysis) were visualized as a network (Figure 6). The network was generated by use of cytoscape (with a fold change ≥2 or ≤0.5, *P* < 0.05), miRanda target prediction, and negative-correlation filtering, revealing that numerous mRNAs including IGF1 mRNA were negatively regulated by the five miRNAs, which were downregulated, thereby increasing the expression of the corresponding mRNA.

### 3.10. IGF1 Protein Quantitation

**Figure 7:**
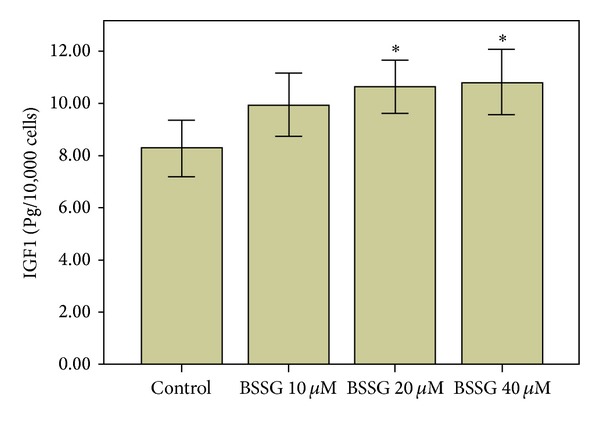
IGF1 protein quantitation. IGF1 protein levels were reported in pg/10,000 cells. IGF1 protein levels were significantly increased after the NSCs were treated with BSSG at 20 and 40 *μ*M. Data are presented as Mean ± SD (*n* = 6); *P < 0.05 compared with the control.

IGF1 protein in the cell culture supernatant was quantified. IGF1 protein levels were remarkably increased after the NSCs were treated with BSSG at 20 and 40 *μ*M (*P* < 0.05, Figure 7).

### 3.11. PPP Inhibition Test

**Figure 8:**
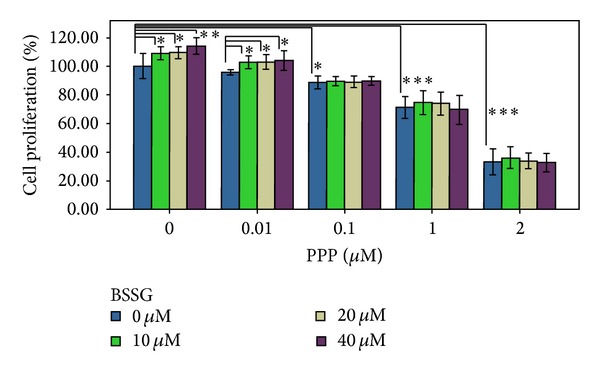
PPP inhibited the BSSG-induced cell proliferation. The inhibition of PPP on NSC proliferation was weak at 0.01 *μ*M; however, the inhibition was remarkable at 0.1 *μ*M (*P* < 0.05) as well as 1 (*P* < 0.001) and 2 *μ*M (*P* < 0.001). Analogously, the inhibition of PPP on BSSG-induced cell proliferation was weak at 0.01 *μ*M. By contrast, BSSG-induced cell proliferation was inhibited when the cells were exposed to PPP ≥ 0.1 *μ*M. This result showed that if the function of IGF1 was blocked up; then the cell proliferation induced by BSSG ceased to exist. Data are presented as Mean ± SD (*n* = 4). *P < 0.05, **P < 0.01, and ***P < 0.001.

PPP inhibited the BSSG-induced cell proliferation at suitable concentrations (Figure 8). The inhibition of PPP on NSC proliferation was weak at 0.01 *μ*M; however, the inhibition was remarkable at 0.1 *μ*M (*P* < 0.05) as well as 1 (*P* < 0.001) and 2 *μ*M (*P* < 0.001). Analogously, the inhibition of PPP on BSSG-induced cell proliferation was weak at 0.01 *μ*M. By contrast, BSSG-induced cell proliferation was inhibited when the cells were exposed to PPP ≥ 0.1 *μ*M. This result showed that if the function of IGF1 was blocked up, then the cell proliferation induced by BSSG ceased to exist.

## 4. Discussion

NSC proliferation is necessary to facilitate neurogenesis [26, 27]. Neurogenesis is enhanced when NSC proliferation is promoted appropriately. The present study provided an efficient alternative substance BSSG, which is inexpensive and chemically stable (BSSG powder remains stable at room temperature for a maximum of two years).

BSSG is present in many higher plants [28]. The procedure of isolation of BSSG is simple [29]. Moreover, it had been synthetically prepared since 1913 [30]; therefore, BSSG can be easily obtained. In the study, the minimal effective concentration was 5 *μ*M, and the effectiveness of BSSG was similar to that of bFGF (20 ng/mL) and EGF (20 ng/mL) at concentrations of 40 *μ*M, revealing the excellent proliferation-promoting activity.

The proliferation-promoting activity of BSSG is the result of regulation of numerous genes. mRNA expression profiling revealed that the majority of upregulation genes were involved in mitotic cell cycle particularly in the M phase, enhancing cell proliferation. By contrast, the majority of the downregulated expression genes were involved in cell differentiation, indicating that cell differentiation was inhibited and accordingly, more possibility of cell proliferation was afforded. Among the differential expression genes, the “hubs” of the gene nodes were Bub1b, Cdc20, Plk1, Spp1, Aurkb, IGF1, and Ndc80, all of which were upregulated. Most of these “hub” genes are necessary to complete a cell cycle according to Genecards (http://www.genecards.org/), of which the importance is self-evident. However, the primary regulatory gene that induces the cells to enter the cell cycle or accelerate the course of the cell cycle is more substantial to cell proliferation. IGF1 is the key regulatory gene in these “hub” genes, since it exhibits a notable growth-promoting activity.

IGF1 gene encodes a protein with functions and structure similar to insulin, but IGF1 exhibits a higher growth-promoting activity. Experimental evidence demonstrates that IGF1 functions in CNS development by promoting neural cell proliferation, survival, and differentiation, in which IGF1 likely functions in a paracrine or autocrine fashion [31]. In vivo activities of IGF1, including the stimulation of NSC and progenitor cell proliferation during embryonic and early postnatal development, have been examined [32]. Moreover, endogenous IGF1 serves an important role in dentate granule cell survival during the course of postnatal brain development [33]. Also, IGF1 is an endogenous mediator of focal ischemia-induced neural progenitor proliferation [34]. BSSG increases the expression of endogenous IGF1 with even more biological activities, since IGF1 plays important role in growth and development of NSCs.

BSSG increased the expression of endogenous IGF1 as showed in mRNA expression microarray analysis, miRNA-mRNA correlation analysis and IGF1 Protein Quantitation. In particular, IGF1 mRNA was negatively regulated by miRNAs (miR-129-5p, miR-301a-3p, and miR-322-5p), which were downregulated after NSCs were treated with BSSG. In other words, the expression of IGF1 mRNA was increased. And as a result, the IGF1 protein level was increased. Emerging evidence supported the close relationship between the abilities of BSSG and IGF1 to promote cell proliferation. PPP inhibition test confirmed that the function of BSSG depended on IGF1, in which the function was inhibited when the cells were exposed to suitable doses of PPP.

The present study provided information about BSSG, an inexpensive and stable compound, which could promote NSC proliferation. BSSG could be potentially developed as a growth factor alternative that could be used in clinical medicine and research applications.

## Figures and Tables

**Table 1:** Primer sequences of the reference gene and the genes selected.

Gene	Primer sequence	Annealing temperature (°C)	Product length (bp)
GAPDH	F: 5′-GGAAAGCTGTGGCGTGAT-3′	60	308
	R: 5′-AAGGTGGAAGAATGGGAGTT-3′		
IGF1	F: 5′-CTGGCACTCTGCTTGCTCAC-3′	60	180
	R: 5′-CTCATCCACAATGCCCGTCT-3′		
cdkn1c	F: 5′-CCTCCCGTTCCCTTCTTTCT-3′	60	96
	R: 5′-CGTTCCATCGCTGTTCTGC-3′		
Espl1	F: 5′-TGACTACCTGGGCGTGACTG-3′	60	98
	R: 5′-CTGGCTCTGAGATGGCACAA-3′		
Pttg1	F: 5′-TGGAGACAGTTGTTTGGGTGC-3′	60	270
	R: 5′-GCTGCCTGGCTCTTCGTTAT-3′		
Ptpru	F: 5′-ACCCTGAGCGAGAACGACA-3′	60	285
	R: 5′-GGGATGGCTGAATAGCAAGAT-3′		

To validate the mRNA expression profiling, five genes (Igf1, cdkn1c, Espl1, Pttg1, and Ptpru) were selected to performed real time-PCR. Primer sequences of the GAPDH gene and the genes selected were listed in Table 1. Igf1: Insulin-like growth factor-1; cdkn1c: cyclin-dependent kinase inhibitor 1C; Espl1: extra spindle pole bodies homolog 1, Pttg1: pituitary tumor-transforming 1; Ptpru: protein tyrosine phosphatase, receptor type, U.

**Table 2:** RT Primer sequence of the internal control gene and the target genes for cDNA synthesis.

Genes	RT primer sequence
U6	5′-CGCTTCACGAATTTGCGTGTCAT-3′
rno-miR-129-5p	5′-GTCGTATCCAGTGCGTGTCGTGGAGTCGGCAATTGCACTGGATACGACGCAAGCC-3′
rno-miR-322-5p	5′-GTCGTATCCAGTGCGTGTCGTGGAGTCGGCAATTGCACTGGATACGACTCCAAAA-3′
rno-miR-301a-3p	5′-GTCGTATCCAGTGCGTGTCGTGGAGTCGGCAATTGCACTGGATACGACGCTTTG-3′

To validate the miRNA expression profiling, three genes were selected to performed real time-PCR. RT primer sequences of the U6 gene used as reference and the genes selected were listed in Table 2.

**Table 3:** Primer sequences of the internal control gene and the target genes for PCR.

Genes	Primer sequence	Annealing temperature (°C)	Product length (bp)
U6	F: 5′GCTTCGGCAGCACATATACTAAAAT3′	60	89
	R: 5′CGCTTCACGAATTTGCGTGTCAT3′		
rno-miR-129-5p	GSP: 5′GGAACTTTTTGCGGTCTGG3′	60	63
	R: 5′GTGCGTGTCGTGGAGTCG3′		
rno-miR-322-5p	GSP: 5′GGGCAGCAGCAATTCAT3′	60	65
	R: 5′CAGTGCGTGTCGTGGAG3′		
rno-miR-301a-3p	GSP: 5′CCCCGTGCAATAGTATTGT3′	60	65
	R: 5′CAGTGCGTGTCGTGGAGT3′		

Primer sequences of the internal control gene and the target genes for PCR were listed in Table 3. GSP is the specific primer for the corresponding miRNA and R is the matching primer for the corresponding RT Primer.

**Table 4:** Upregulation genes.

GO ID	Term	*P* value	Genes
GO:0000087	M phase of the cell cycle	9.58*E* − 09	AURKB; CCNF; KIF2C; BUB1B; CDCA3; IGF1; CENPF; DLGAP5; CDC20; SPAG5; MAD2L1; TRAF4AF1; NUSAP1; ESPL1; CCNB1; PLK1; PTTG1
GO:0000278	Mitotic cell cycle	8.23*E* − 08	SPAG5; MAD2L1; TRAF4AF1; NUSAP1; ESPL1; CDKN1B; CENPF; DLGAP5; CENPA; NDC80; CCNB1; PTTG1; CDC20; AURKB; CCNF;
GO:0000280	Nuclear division	1.82*E* − 07	SPAG5; MAD2L1; TRAF4AF1; NUSAP1; ESPL1; CCNB1; PLK1; PTTG1; CDC20; AURKB; CCNF; KIF2C; BUB1B; CDCA3; IGF1
GO:0007059	Chromosome segregation	4.12*E* − 07	SPAG5; MAD2L1; TRAF4AF1; NUSAP1; ESPL1; CCNB1; NDC80; PTTG1; CENPF; KIF2C; TOP2A
GO:0048285	Organelle fission	4.86*E* − 07	SPAG5; MAD2L1; TRAF4AF1; NUSAP1; ESPL1; CCNB1; CDCA3; PLK1; PTTG1; CDC20; AURKB; CCNF; KIF2C; BUB1B; IGF1
GO:0000226	Microtubule cytoskeleton organization	1.87*E* − 06	CENPA; NDC80; ESPL1; PLK1; KIF20A; KIF2C; SPAG5; TEKT1; TRAF4AF1; CCNF; TACC3; CDKN1B; NUSAP1; CCNB1; AURKB
GO:0048545	Response to steroid hormone stimulus	2.36*E* − 06	CAR9; GBA; A2M; HP; IGF1; ADM; and so forth, a total of 22 genes
GO:0051301	Cell division	3.80*E* − 06	NUSAP1; PLK1; AURKB; KIF20A; NUMBL; TOP2A; TXNIP; CCNB2; CCNB1; PTTG1; CDC20; CCNF; KIF2C; BUB1B; CDCA3; TRAF4AF1
GO:0008283	Cell proliferation	6.93*E* − 06	CDC20; AURKB; CCNB1; PTTG1; IGF1; and so forth, a total of 39 genes
GO:0019932	Second messenger-mediated signaling	1.12*E* − 05	CALCA; ADORA2A; GRM3; EDNRB; GRM5; CXCR4; TOX3; RASD1; ADM; PDE7B; CDH13; IGF1; LMCD1; MT1A
GO:0009605	Response to external stimulus	1.25*E* − 05	A2M; LBP; CCNB1; ENPP2; CKLF; IGF1; and so forth, a total of 35 genes
GO:0009719	Response to endogenous stimulus	1.94*E* − 05	ADORA2A; IGF1; SPP1; A2M; and so forth, a total of 32 genes
GO:0008608	Attachment of spindle microtubules to kinetochores	2.25*E* − 05	CCNB1; SPAG5; TRAF4AF1; NDC80
GO:0009056	Catabolism	2.78*E* − 05	TOP2A; FBXO32; MANBA; IGF1; CDC20; and so forth, totle 41 genes
GO:0007051	Spindle organization	3.11*E* − 05	ESPL1; NDC80; TACC3; CCNB1; AURKB; SPAG5; TRAF4AF1
GO:0051313	Attachment of spindle microtubules to chromosomes	4.44*E* − 05	NDC80; CCNB1; SPAG5; TRAF4AF1
GO:0009725	Response to hormone stimulus	5.00*E* − 05	IGF1; LOX; SPP1; A2M; HP; ALPL; and so forth, a total of 27 genes
GO:0010941	Regulation of cell death	2.39*E* − 04	AURKB; IGF1; ADORA2A; and so forth, a total of 32 genes
GO:0043470	Regulation of carbohydrate catabolism	8.83*E* − 04	PFKFB3; DDIT4; IER3; IGF1
GO:0048016	Inositol phosphate-mediated signaling	1.28*E* − 03	EDNRB; GRM5; CALCA; IGF1; LMCD1

Upregulation genes obtained from the mRNA expression profiling were listed in Table 4. The upregulation genes were mostly involved in the mitotic cell cycle, enhancing cell proliferation. *P* value, the significance testing value of the GO ID, results from the top GO of a bioconductor.

**Table 5:** Downregulation genes.

GO ID	Term	*P* value	Genes
GO:0030154	Cell differentiation	3.02*E* − 06	FOXC2; IGF2; JAG1; SEMA3C; HMGA2; and so forth, a total of 38 genes
GO:0006950	Response to stress	2.05*E* − 04	PENK; BDNF; CRYAB; TRH; PLAU; MMP3; and so forth, a total of 32 genes
GO:2000736	Regulation of stem cell differentiation	4.89*E* − 04	HMGA2; JAG1; HES1
GO:0032103	Positive regulation of response to external stimulus	6.01*E* − 04	NPY; IL1RL1; SCG2; TNFSF11; CD74; THBS4
GO:0048710	Regulation of astrocyte differentiation	8.72*E* − 04	HES1; CLCF1; HMGA2
GO:0045597	Positive regulation of cell differentiation	1.16*E* − 03	TGFB1I1; CD74; FRZB; JAG1; BDNF; MAP1B; IFI204; TNFSF11; TNFRSF12A; HES1; CLCF1
GO:0048584	Positive regulation of response to stimulus	2.06*E* − 03	CD74; TNFSF11; TGFB1I1; CDKN1C; GPC3; HES1; NPY; CLCF1; IGF2; JAG1; PRRX2; IL1RL1; SCG2; THBS4; HMGA2; TNFRSF12A
GO:0000904	Cell morphogenesis involved in differentiation	5.26*E* − 03	HMGA2; MAP1B; BDNF; TGFB1I1; CHST3; FOXC2; TNFRSF12A; HES1; XYLT1; NPTX1
GO:0050920	Regulation of chemotaxis	6.38*E* − 03	SCG2; CD74; THBS4; EFNB2
GO:0030182	Neuron differentiation	7.29*E* − 03	MAP1B; BDNF; CHST3; NPY; MFRP; JAG1; HES1; CDKN1C; TNFRSF12A; HCN1; XYLT1; THBS4; NPTX1; BYSL
GO:0060326	Cell chemotaxis	1.53*E* − 02	TNFSF11; SCG2; CD74; THBS4
GO:0090398	Cellular senescence	1.67*E* − 02	HMGA2; RGD1305645
GO:0016477	Cell migration	2.55*E* − 02	SEMA3C; EFNB2; TNFSF11; TNFRSF12A; PLAU; MMP3; UNC5C; SCG2; THBS4; CD74; HES1
GO:0016126	Sterol biosynthesis	4.53*E* − 02	HMGCS2; HSD17B7
GO:0033554	Cellular response to stress	4.91*E* − 02	HMGA2; TNFSF11; MAP1B; CHST3; XYLT1; DHX9

Downregulation genes obtained from the mRNA expression profiling were listed in Table 5. The downregulation genes were mostly involved in differentiation and the regulation of differentiation, indicating that cell differentiation was inhibited, and accordingly, more possibility of cell proliferation was afforded. *P* value, the significance testing value of the GO ID, results from the top GO of a bioconductor.

**Table 6:** Comparison of quantified mRNA expressions obtained using real-time PCR and microarray analysis.

Genes	PCR		Microarray analysis		Fold change (test versus control)	
	Control	Test	Control	Test	PCR	Microarray analysis
IGF1	0.061 ± 0.0043	0.196 ± 0.0098	230.514 ± 49.286	1079.355 ± 144.074	3.20 ↑	4.75 ↑
Pttg1	0.025 ± 0.0007	0.051 ± 0.007	319.160 ± 31.644	931.231 ± 116.161	2.09 ↑	2.91 ↑
Adora2a	0.053 ± 0.0028	0.125 ± 0.025	590.370 ± 64.092	1544.692 ± 112.533	2.34 ↑	2.62 ↑
Espl1	0.008 ± 0.002	0.028 ± 0.008	349.823 ± 33.064	730.605 ± 135.345	3.41 ↑	2.07 ↑
Ptpru	0.028 ± 0.010	0.078 ± 0.011	537.963 ± 83.725	1321.925 ± 187.830	2.74 ↑	2.46 ↑
cdkn1c	0.058 ± 0.028	0.018 ± 0.017	2536.464 ± 115.441	1196.195 ± 188.255	0.30 ↓	0.47 ↓

**Table 7:** Comparison of the quantified miRNA expressions obtained by real-time PCR and microarray analysis.

miRNA	PCR		Microarray analysis		Fold change (test versus control)	
	Control	Test	Control	Test	PCR	Microarray analysis
rno-miR-129-5p	1.00 ± 0.055	0.29 ± 0.090	0.88 ± 0.17	0.32 ± 0.08	0.29 ↓	0.36 ↓
rno-miR-301a-3p	0.89 ± 0.11	0.29 ± 0.12	3.47 ± 1.15	1.08 ± 0.27	0.33 ↓	0.31 ↓
rno-miR-322-5p	1.34 ± 0.33	0.46 ± 0.15	1.95 ± 0.55	0.73 ± 0.11	0.34 ↓	0.37 ↓
